# The Heart of the Matter: Immune Checkpoint Inhibitors and Immune-Related Adverse Events on the Cardiovascular System

**DOI:** 10.3390/cancers15245707

**Published:** 2023-12-05

**Authors:** Chase E. Green, Jessica Chacon, Brandon M. Godinich, Rivers Hock, Maria Kiesewetter, Mark Raynor, Komal Marwaha, Satish Maharaj, Nathan Holland

**Affiliations:** 1 Department of Medical Education, Paul L. Foster School of Medicine, Texas Tech Health Sciences Center El Paso, 5001 El Paso Ave., El Paso, TX 79905, USA; 2 Department of Internal Medicine, Division of Hematology/Oncology, Paul L. Foster School of Medicine, Texas Tech Health Sciences Center El Paso, 4800 Alberta Ave., El Paso, TX 79905, USA

**Keywords:** cardiovascular, anti-PD-1, anti-CTLA-4, immunotherapy, immune-related adverse event

## Abstract

**Simple Summary:**

Cancer remains one of the leading killers world-wide. In recent years new drugs to treat cancer that exploit the immune system to attack cancer have been developed called immune checkpoint inhibitors (ICIs). As use of these potent anti-cancer therapeutics have grown, researchers have noticed an unsettling association with use of ICIs and cardiovascular complications known as immune-related adverse events (irAEs). This review discusses how ICIs work as a cancer treatment, how to clinically recognize and manage patients with cardiovascular irAEs, what risk factors may contribute to irAEs, and discusses some of the current theories of mechanisms driving cardiovascular irAEs.

**Abstract:**

Cancer remains a prominent global cause of mortality, second only to cardiovascular disease. The past decades have witnessed substantial advancements in anti-cancer therapies, resulting in improved outcomes. Among these advancements, immunotherapy has emerged as a promising breakthrough, leveraging the immune system to target and eliminate cancer cells. Despite the remarkable potential of immunotherapy, concerns have arisen regarding associations with adverse cardiovascular events. This review examines the complex interplay between immunotherapy and cardiovascular toxicity and provides an overview of immunotherapy mechanisms, clinical perspectives, and potential biomarkers for adverse events, while delving into the intricate immune responses and evasion mechanisms displayed by cancer cells. The focus extends to the role of immune checkpoint inhibitors in cancer therapy, including CTLA-4, PD-1, and PD-L1 targeting antibodies. This review underscores the multifaceted challenges of managing immunotherapy-related cardiovascular toxicity. Risk factors for immune-related adverse events and major adverse cardiac events are explored, encompassing pharmacological, treatment-related, autoimmune, cardiovascular, tumor-related, social, genetic, and immune-related factors. The review also advocates for enhanced medical education and risk assessment tools to identify high-risk patients for preventive measures. Baseline cardiovascular evaluations, potential prophylactic strategies, and monitoring of emerging toxicity symptoms are discussed, along with the potential of adjunct anti-inflammatory therapies.

## 1. Introduction

Cancer remains one of the leading causes of death worldwide, second only to cardiovascular disease. Over the past decades, advancements in anti-cancer therapies have increased the likelihood of positive outcomes and reduced morbidity and mortality for many types of cancer. Immunotherapy, one of the most promising recent developments in anti-cancer therapy, uses the immune system to target and destroy cancer. Despite the immense promise of this class of anti-cancer therapeutics, concerns have been raised regarding the association between immunotherapy and adverse cardiovascular events. These concerns have led to the investigation of potential cardiovascular toxicity resulting from the use of immunotherapy. This review will provide an overview of how immune checkpoint inhibitors work, clinical perspectives on checkpoint inhibitors, potential risk factors and biomarkers for monitoring and anticipating adverse events, as well as a discussion of possible mechanisms that may drive the development of immune-related adverse events (irAE), particularly major cardiac adverse events (MACE) as well as vasculopathy.

## 2. Introduction to Immunotherapy

### 2.1. Immune Response to Cancer Cells

Once a dendritic cell (DC) encounters an antigen, the DC processes and presents the antigen to T cells in the lymph node (LN). For a T cell to become activated, the DC must present the antigen via the peptide: MHC class I/II complex interacting with the T-cell Receptor (TCR) on the T cell (signal 1) [1]. After this interaction, CD28 on the T cell binds to the B7 family (CD80/CD86) on the DC to provide a co-stimulatory signal (signal 2). Once co-stimulation occurs, cytokines, such as IL-12, are then released by the DC to activate the T-cell (signal 3) fully. The activated T cells then exit the LN and travel to the site of infection or tumor site, where the T cell will provide help in the context of a T helper (Th) cell or induce cytotoxic effects in the context of a CD8+ cell [2]. Another example of the immune response against cancer cells is through Natural Killer (NK) cells. NK cells are innate immune cells that induce a cytotoxic effect on their target using perforin and granzymes, similar to CD8+ T cells. However, NK cells can recognize and kill their target cells in an MHC-independent manner [3]. M1 macrophages exhibit anti-tumor effects via intrinsic phagocytosis and release Reactive Oxygen Species (ROS) and Nitric Oxide (NO), which have cytotoxic effects on tumor cells [3].

### 2.2. Mechanisms of Tumor Immune System Evasion

Although the immune system has anti-tumor effects, cancer cells can also adapt and evade the immune system. Some examples of immune cells that contribute to cancer cell growth and survival are T regulatory (Treg) cells, M2 macrophages, and myeloid-derived suppressor cells (MDSC) [3]. Tregs are an immunosuppressive subset of CD4+ cells that can contribute to tumor growth using various approaches. Specifically, Tregs constitutively express CTLA-4, thereby inhibiting co-stimulatory signals by CD80/CD86. They also secrete IL-2, providing a proliferative and survival autocrine signal, and they secrete inhibitory cytokines, such as IL-10 and TGF-b, inducing the development of tolerogenic DCs within the Tumor Microenvironment (TME) [3]. In addition, Tregs express PD-1 and PD-L1, further inhibiting the immune response within the TME. Tumor cells can also escape recognition by T cells by downregulating MHC-I and exhibiting antigen loss [3].

### 2.3. Role of Immune Checkpoint Inhibitors in Cancer Therapy

Immunotherapy is a type of therapy that utilizes the immune system to treat cancer. Immunotherapy can be categorized as passive or active. Passive immunotherapy targets the tumor and involves administering immune-cell factors, resulting in an immediate effect [4]. Because the immunological memory is not initiated, continued dosing may be required for passive immunotherapy. Active immunotherapy acts directly on the immune system, induces an immunological memory, and produces a lasting, durable response [4]. Examples of passive and active immunotherapies include DC immunotherapy, vaccines, monoclonal antibodies (mAb) that target tumor-specific or overexpressed antigens and cytokines (e.g., IL-2, IFN-g, and TNF-a), and Adoptive Cell Therapies (ACT) (e.g., Tumor-Infiltrating Lymphocyte (TIL) therapy and Chimeric Antigen receptor (CAR) T cells) [5].

**Figure 1:**
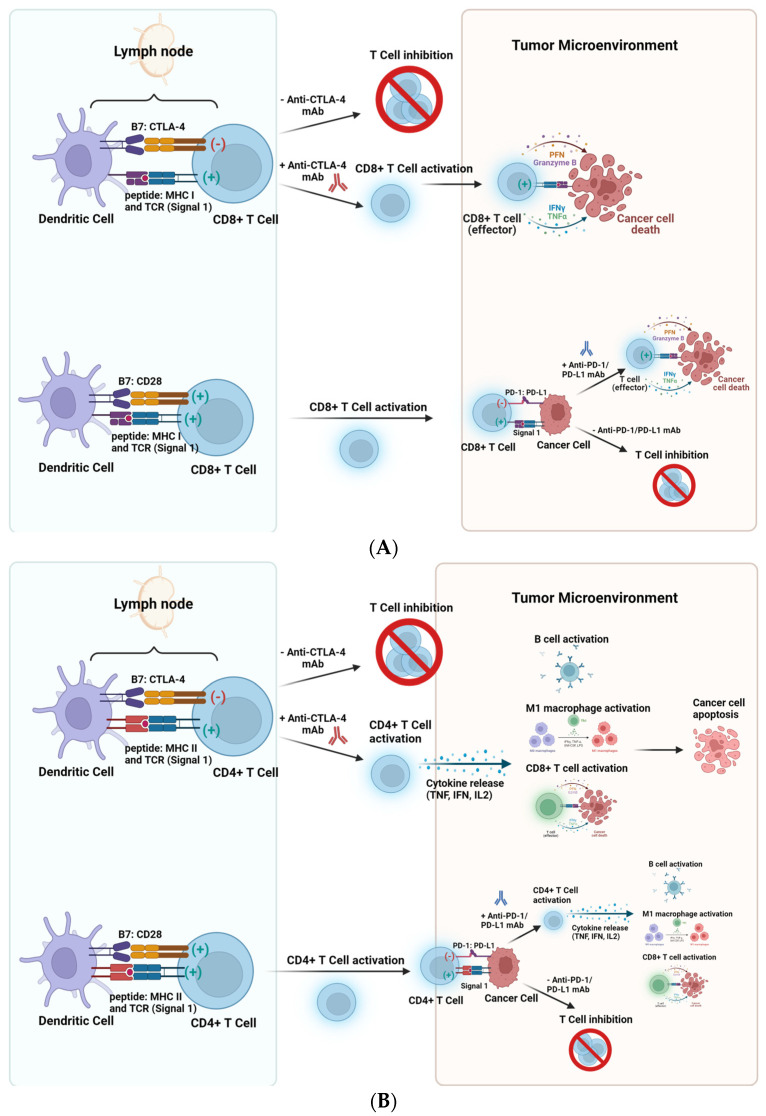
Approaches using anti-CTLA-4 mAb, anti-PD-1 mAb, and anti-PD-L1 mAb in cancer therapy. (**A**) (**Top**): CTLA-4 competes with CD28 to bind to CD80 or CD86 and engagement of CTLA-4 with CD80/CD86 results in dampened immune responses. Ipilimumab (anti-CTLA-4 mAb) prevents CD8+ T-cell attenuation at the priming phase, allowing the expansion of effector T cells. (**A**) (**Bottom**): Upon binding of the TCR with peptide, MHC I (signal 1) and the engagement of B7 (CD80/CD86) with CD28 (signal 2), CD8+ T cell activation occurs. Within the Tumor Microenvironment, cancer cells can express PD-L1 and bind to PD-1 on T cells, leading to T cell inhibition. However, blocking this engagement using anti-PD-1/anti-PD-L1 mAbs enables CD8+ T cell activation and subsequent cancer cell death. (**B**) (**Top**): CTLA-4 competes with CD28 to bind to the B7 family and engagement of CTLA-4 with CD80/CD86 results in dampened CD4+ T cell responses. Anti-CTLA-4 mAb prevents CD4+ T-cell attenuation at the priming phase, allowing the expansion of CD4+ T cells. (**B**) (**Bottom**): Upon binding of the TCR with peptide, MHC II (signal 1) and the engagement of B7 (CD80/CD86) with CD28 (signal 2), CD4+ T cell activation occurs, resulting in cytokine secretion and immune cell activation. Within the Tumor Microenvironment, cancer cells can express PD-L1 and bind to PD-1 on T cells, leading to CD4+ T cell inhibition. However, blocking this engagement using anti-PD-1/anti-PD-L1 mAbs enables B cell activation, M1 macrophage polarization and CD8+ T cell activation and subsequent cancer cell death. (Figure 1 was created using Biorender).

Cancer cells can evade the immune system by upregulating inhibitory receptors and ligands on their cell surface, such as CTLA-4, PD-1, PD-L1/2, Lag-3, and Tim-3 [6]. Immune checkpoint Inhibitors (ICI) are another category of cancer immunotherapy. ICIs block immune checkpoint proteins from binding with partner proteins. This review will primarily focus on three types of ICIs: anti-CTLA-4 mAb, anti-PD-1 mAb, and anti-PD-L1 mAb. Figure 1 illustrates the approaches in CD8+ and CD4+ T cell responses using these ICIs in cancer therapy.

Upon the T cell priming phase, CTLA-4 competes with CD28 to bind to CD80 or CD86. CTLA-4 has a higher affinity for CD80/CD86 than CD28 and the subsequent binding of CTLA-4 to the B7 family (CD80/CD86), results in immune suppression. The ligation of CTLA-4 with the B7 family is one approach that tumor cells use to evade the immune response. Therefore, interfering with this engagement was crucial for the activation of the immune system. In 2011, the United States Food and Drug Administration (FDA) approved an anti-CTLA-4 antibody (Ipilimumab) [7,8]. Ipilimumab prevents the binding of CTLA-4 to the B7 family, allowing the immune system to become activated at the priming phase, activating and expanding effector T cells. This initial FDA approval led to the investigation and development of other checkpoints, such as PD-1 and PD-L1, that could be targeted. Engagement of PD-1 to its ligand, PD-L1, results in innate and adaptive immune suppression [6]. In 2014, nivolumab was the first anti-PD-1 blocking antibody to be FDA-approved [9]. In a Phase III clinical trial, nivolumab, compared with dacarbazine (a chemotherapy drug), achieved overall response rates (ORR) of 40% vs. 13.9%, with 1-year survival rates of 72.9% vs. 42.1% [9]. Another anti-PD-1 antibody that was approved by the FDA in 2014 is Pembrolizumab. Melanoma patients who received Pembrolizumab exhibited an ORR of 52% with 25% complete remission (CR).

The clinical effects of blocking inhibitory receptors as monotherapy lead to investigating combination approaches for cancer treatment. In metastatic melanoma patients, administering nivolumab and ipilimumab resulted in increased response rates and survival [10,11]. Specifically, the combination therapy resulted in a 52% overall survival at 5 years, exhibiting improved efficacy compared with either single agent alone. The promising results of these combinatory agent studies subsequentially led to the FDA approval of nivolumumab and ipilimumab for the treatment of different cancer types, including non-small cell lung cancer, unresectable malignant pleural mesothelioma, and intermediate or poor-risk advanced renal cell carcinoma. However, the increased anti-tumor efficacy of nivolumumab and ipilimumab combination therapy has been associated with increased immune-related toxicity [10,11].

Under normal conditions, the role of PD-1 and CTLA-4 is to prevent autoimmunity and limit immune activation to prevent bystander damage. Blocking these inhibitory receptors through therapeutic antibodies for cancer treatment has been associated with a wide range of side effects that resemble autoimmune reactions. This review article will focus on the immune-related adverse events seen in the cardiovascular system after the administration of ICIs.

## 3. Mechanisms of Cardiovascular Injury

A growing body of clinical evidence demonstrates a mechanistic association between immunotherapy and averse cardiovascular events. However, due to the various types of injuries or acceleration of underlying pathological cardiovascular conditions, it is unlikely that there is one putative mechanism which drives cardiovascular injury in immunotherapy. Just as there are several manifestations of irAEs affecting the heart and vasculature, there are several potential mechanisms hypothesized which may drive injury during or following treatment with immunotherapy, including checkpoint inhibitors.

### 3.1. Mechanisms of Cardiac Injury

Cardiac injury following immunotherapy, particularly with checkpoint inhibitors, may result from the unchecked activity of immune cells interacting with cardiac tissue. It has been known for almost three decades that inhibition of immune checkpoints can promote inappropriate lymphocytic targeting of healthy, non-cancerous tissue. CTLA-4 deficient mice have been shown to develop cardiac lymphocyte infiltration, resulting in lethal myocarditis [12]. Another study conducted on mice with generated CTLA4-/- cells showed a large increase in granulocyte–macrophage colony-stimulating factor (GM-CSF), IL-4, and IFN-g. This was a large increase compared to mice with normal CTLA-4 expression. These mice developed severe myocarditis [12]. This study again points to the importance of CTLA-4 in homeostasis in cardiac tissue. Individuals deficient in this regulator or those receiving immunotherapy targeting this regulator will be at risk. Measuring increasing levels of these cytokines may provide a useful way to gauge if the patient is at risk for myocarditis.

CTLA-4 is not the only protein receptor to be experimentally implicated in the development of cardiac compilations. PD-1 knockout models in mice have demonstrated dilated cardiomyopathy following the development of autoantibodies to cardiac troponin I or potentially other cardiac proteins, such as myosin or b1 adrenergic receptors [13]. Targeting these proteins by self-antigens could result in inappropriate activation of voltage-gated L-type Ca++ channels, resulting in myocardial stress. However, this finding is questioned, as similar PD-1-deficient BALB/c mice did not develop spontaneous dilated cardiomyopathy [14]. Heart tissue of patients who died of myocarditis following therapy with PD-1 inhibitors revealed T cell receptors identical to those found in cloned T cells in tumors, as well as the tumors demonstrating striated muscle-specific antigens [15]. The involvement of PD-1 suppression in developing cardiac dysfunction and injury is further supported experimentally in mice. Upregulation of PD-1 by IFN-g in a cytotoxic T-cell-induced myocarditis model was cardioprotective [16], suggesting that loss of PD-1 may increase susceptibility to T-cell-mediated cardiac injury. Interestingly, knockout of PD-1 in mice was not associated with myocardial infiltration of immune cells [17], unlike CTLA-4 deficient mice. Excessive activation of cytokine release and subsequent immune dysregulation has been proposed as a mechanism that could explain ICI-induced MACE and cardiac injury; however, cytokine release syndrome is more often associated with the usage of CAR T cell therapy [18]. Despite this, it is known that pro-inflammatory cytokines, including TNF-a, IL-6, and IL-12, can lead to cardiotoxicity through various mechanisms, including dysregulated b-adrenergic signaling [19] and their role cannot be discounted.

### 3.2. Mechanisms of Vascular Injury

It is also important to consider vascular injury when considering the risk of adverse cardiovascular events and the underlying mechanisms that may drive irAE. A retrospective review found that patients had an increased risk of adverse vascular events (AVEs) when receiving ICI therapy like those receiving chemotherapy. They concluded that caution should be exercised for patients who already show risk factors of having AVEs [20]. The investigators hypothesized that baseline inflammatory state, as measured by a neutrophil-to-lymphocyte ratio (NLR) and C-reactive protein (CRP), would be elevated in patients with AVEs compared to controls [20]. The results from the study indicated baseline NLR was elevated in patients who developed AVEs compared to those without MACE or other irAEs. However, there were no differences noted between baseline CRP concentrations and the authors concluded that NLR may be useful in the screening and diagnosing of AVEs in cancers treated with ICIs.

Perhaps most concerning is the potential for ICIs to accelerate the development of atherosclerosis. Atherosclerosis is the accumulation of lipid-containing cells underneath large artery endothelium [21]. Specifically, endothelial macrophages and helper T cells are recruited across the arterial wall via leukocyte adhesion molecules E-selectin, intracellular adhesion molecule-1 (ICAM-1), and vascular adhesion molecule-1 (VCAM-1) by way of NF-kB regulation [22]. Monocytes, or macrophages, then phagocytose many low-density lipoproteins (LDL) utilizing a multitude of receptors, including oxLDL, and oxidize the lipid, killing the macrophage and inducing a metamorphosis into a foam cell [21,22]. Once a foam cell forms, neighboring T lymphocytes are hypothesized to encourage lesion progression via cytokine release, such as IL-9, IL-11, and IL-12, increasing the plaque size [22]. After a long enough period of plaque expansion and foam cell accumulation, the atheroma, or atherosclerotic plaque, contains a lipid or necrotic core covered by a layer of fibrotic cap consisting of both smooth muscle cells and extracellular matrix [21]. The fate of this plaque is determined by the stability or thickness of the base of the atherosclerotic lesion, which contains foam cells and T lymphocytes [21]. A stable plaque with a thick enough shoulder to prevent disruption of the necrotic core will likely remain clinically silent until the plaque occludes the vascular lumen enough to cause arterial stenosis [21]. However, a vulnerable plaque with a weak shoulder is potentially fatal due to plaque rupture contributing to ACS or thrombosis [21].

In 2020, a revolutionary clinical study discovered that ICI treatment proposes an increased risk of atherosclerotic cardiovascular events [23]. According to prior murine-based research, PD-1, PD-L1, and CTLA-4 serve as negative regulators of atherosclerosis, blocking the ability for T lymphocytes to upregulate macrophages within arterial walls; therefore, while treating cancerous lesions in the body, immune checkpoint inhibitors allow promotion of monocytic antigen presentation and T lymphocyte proliferation within atherosclerotic plaque lesions [24,25]. A proposed mechanism of upregulating atherosclerosis may be creating the proper microenvironment to promote immune crosstalk between T lymphocytes and APCs.

In fact, during observation of patients with melanoma being treated with ICI therapy, a four-fold increase in risk for MI, coronary revascularization, and ischemic stroke was observed [23]. Additionally, a three-fold increased total plaque volume progression rate, from 2.1 to 6.7, was observed in calcified and non-calcified atherosclerotic plaques [23]. In terms of how the atherosclerosis acceleration occurs, a murine model demonstrated that PD-1-deficient myeloid progenitor cells upregulated genes involved in cholesterol synthesis and uptake, and downregulated genes promoting cholesterol metabolism, leading to overall markedly increased cellular cholesterol levels, known to increase the risk and rate of atherosclerosis [26]. Therefore, a potential mechanism for ICI-induced acceleration of atherosclerosis may lie in the blockade of PD-1, culminating in the upregulation of a pro-atherosclerotic ecosystem in patients. However, a murine-based model analyzing the effects of ICI treatment on hyperlipidemic mice noted that short-term treatment induces an activated T-cell profile without directly affecting the myeloid system [27]. Specifically, a 3.9-fold increase in plaque necrotic core area with an immunogenic profile of markedly increased cytotoxic CD3+ CD8+ T cells and a 41.9% decrease in plaque macrophage content suggest increased macrophage apoptosis promoted necrotic core formation and subsequent plaque progression [27]. There is additional evidence that dual therapy with anti-CTLA-4/anti-PD-1 induced endothelial activation via increased expression of ICAM-1 and VCAM-1, suggesting upregulation of initial phases in atherosclerosis and the potential for new plaque development in addition to atherosclerotic plaque progression [27]. Therefore, another theoretical mechanism may be the enrichment of cytotoxic T lymphocytes, specifically leading to atherogenesis and a more vulnerable plaque phenotype, placing patients at risk of fatal atherosclerotic complications [25,28].

## 4. Clinical Perspectives of Immunotherapy

### 4.1. Current Clinical Studies in Immunotherapy

Immunotherapy has become the groundbreaking research focus for cancer treatment. Although the immune system can prevent or slow the growth of tumors, cancer cells have developed methods to evade the immune system. This includes, but is not limited to, novel genetic variants that hide cancer cells from the immune system, the addition of proteins on cell surfaces to turn off immune cells, and altering surrounding normal cells that will interfere with immune system responses to cancer cells.

In the clinical context, ICIs can have relatively minimal side effects, including rash, diarrhea, and fatigue [29,30]. The growth of ICIs in the last decade has offered heightened clinical efficacy in patients with cancer. The increased use of ICIs has radically changed the oncology field and offered a more holistic perspective to personalized treatment options [31]. Improved patient outcomes have been seen with advanced anti-cancer therapies, including ICIs and combination approaches. However, several randomized clinical trials have also shown that these treatments may result in severe cardiovascular side effects like heart failure, arrhythmias, thrombotic events, and various heart diseases. While heart failure and myocardial dysfunction are the most concerning, vascular complications are also commonly reported [32]. It is crucial to screen for cardiotoxic effects accurately to ensure patient safety and avoid overly cautious diagnoses that may impede optimal cancer treatment.

### 4.2. Investigation Techniques: Baseline Assessment, ECG, Acute Biomarkers, Imaging

Multiple techniques are employed in investigating suspected cardiac or vascular toxicity, although an overarching stepwise guideline has yet to be established. To properly assess an individual, a baseline assessment of cardiovascular risk, including comprehensive patient history; physical exam; screening for cardiovascular diseases such as coronary artery disease, stroke, or thromboembolic events; and screening for unfavorable lifestyle choices such as tobacco smoking, obesity, alcohol use or physical inactivity [33]. If a proper baseline is established, the following toxicity measurements will be identifiable for the patient’s cardiac or vascular functionality. A summary of common immune-related cardiovascular adverse events and the presentation, diagnosis, and inciting therapeutics is included in Table 1.

Acquisition of an electrocardiogram (ECG) is the first step in most cardiovascular evaluations. In multiple assessments of ICI-induced myocarditis, the most common cardiotoxicity identified as an irAE with ICI therapy, 94.4% of confirmed myocarditis cases showed irregular ECG markings, including abnormal T waves, ST segment, conduction defects, and sinus tachycardia [48]. Another study recorded an immense array of ICI-induced ECG changes, such as prolonged QRS and QTc, decreased QRS voltage, and various conduction blocks [49]. Therefore, there remains an impetus for baseline ECG on patients before ICI treatment to properly assess ECG changes in patients with suspected cardiovascular toxicity.

Acute cardiac biomarkers such as troponin, brain natriuretic peptide (BNP)/proBNP, and LDH may indicate ongoing cardiac adverse effects [50,51,52]. Biomarkers can help detect subclinical LV dysfunction [53]. Troponin, traditionally recognized as a marker of cardiac ischemia, may show prognostic significance in ICI-related myocarditis [49]. Additionally, one meta-analysis noted that troponin levels were increased in 94% of patients with cardiotoxicity, not necessarily myocarditis, showing that troponin may help explore various aspects of cardiac adverse events [40]. Troponin levels have been noted in patients lacking clinically apparent symptoms of cardiac irAEs, which begs the question of smoldering myocarditis or subclinical cardiotoxicity in patients treated with ICIs [40]. Brain natriuretic peptide (BNP)/N-terminal proBNP (NT-proBNP) are very sensitive markers of early myocardial damage [53] An increase in cardiac LDH levels may indicate ICI-induced myocarditis [48]. However, LDH may not distinguish correctly between cases of mild myocarditis versus severe myocarditis [48].

Various imaging studies have been described to investigate ICI-related cardiotoxicity. Echocardiography, nuclear cardiac imaging, specifically multigated radionuclide angiography (MUGA), and cardiac magnetic resonance (CMR) imaging are usually recommended. Multigated acquisition scans and cardiac magnetic resonance (CMR) are more accurate and reproducible than echocardiography for repeated assessments of left ventricular ejection fraction, but due to easy availability, echocardiography is usually used and studies recommend a baseline echocardiogram to provide information on cardiac dysfunction, similarly to baseline ECG [54]. Echocardiograms prove extremely useful in evaluating left ventricular ejection fraction (LVEF) and global longitudinal strain (GLS) [54]. This information shows that ICI-induced cardiovascular toxicity may not always present as symptomatic or clinically apparent, and subclinical cardiotoxicity may not remain subclinical [53].

### 4.3. Importance of Recognition of Cardiovascular irAEs

While myocarditis is the most common cardiotoxicity related to immunotherapy treatment, recent studies indicate that pericardial disease, vasculitis, ACS, arrhythmias, left ventricular dysfunction, and even Takotsubo, or stress, cardiomyopathy may also represent various presentations of ICI-induced cardiotoxicity [49]. While certain MACE, such as myocardial infarction or cardiac arrest, rapidly manifest clinically and are likely to be accounted for in any clinical trial or patient chart, the presence of subclinical cardiac anomalies should not be underestimated nor ignored. Morbidity or mortality rates are likely underestimated due to a lack of investigation concerning cardiac-induced deaths within long-term, extremely ill cancer patients. In a systematic review and meta-analysis, myocarditis mortality could range from 35–50%, and pericardial disease occurs in 0.36% of patients, with a projected mortality of 21%, fourfold higher than patients not treated via immunotherapy [49]. Furthermore, increased troponin was identified in over 50% of ICI-treated patients, indicating the possibility of smoldering myocarditis present in patients without the development of fulminant or clinical myocarditis manifestation, raising the question of patient complications in a long-term setting [40]. Without more stringent guidelines in monitoring and diagnosing MACE within immunotherapy-treated patients, oncologists and patients risk unexpected cardiac complications and death based on current data.

In terms of the gravity concerning thrombotic events as an irAE within immunotherapy-treated cancer patients, multiple studies in clinical settings showed that immunotherapy patients develop more thrombotic events than the general cancer population, including greater pulmonary embolism (PE)-linked mortality [40,55]. Additionally, PE decreased overall survival in those patients who experienced a thrombotic event, whether the event itself led to death or, by an unknown mechanism, the PE provided health complications later in the patient’s life, unrelated to cancer treatment. Furthermore, mortality remained significantly greater in patients who developed VTE than those without VTE events within immunotherapy-treated patients, with additional data suggesting significantly reduced overall survival in VTE-affected immunotherapy patients [56]. Specifically, symptomatic PE posed prognostic significance, suggesting a direct impact of VTE events on mortality in post-operative settings [56]. Additionally, VTE was an independent predictor of early, all-cause mortality, even when investigators adjusted for lab abnormalities in creatinine, alkaline phosphatase, and protein levels [56]. In summary, the potentially life-threatening nature of thrombotic events as an irAE during and after immunotherapy creates an impetus for further investigation and stringent protocols to abate patient mortality.

### 4.4. Cardiovascular Toxicity in Pediatric Patients Treated with Immunotherapy

Immunotherapy is approved for pediatric use, however, there is less experience with immune-related adverse effects in this population. FDA approval of immunotherapy for treating pediatric patients with melanoma has largely been extrapolated from the same treatment approach used in adults. For example, the KEYNOTE-716 phase III trial evaluating the use of adjuvant pembrolizumab in high-risk node-negative melanoma enrolled two pediatric patients (between the ages of 12 and 17 years) [57]. Similarly, atezolizumab was recently approved for the treatment of patients 2 years of age or older with alveolar soft part sarcoma based on results from the ML39345 trial (NCT03141684), in which three pediatric patients were reported [58]. Nivolumab is approved for treatment of pediatric melanoma with results extrapolated from clinical trials of adjuvant nivolumab in adults with melanoma.

The majority of existing data reporting on cardiotxicity in the pediatric population can be derived from four recent phase I/II studies investigating nivolumab (ADVL1412; NCT0230445848), pembrolizumab (KEYNOTE-051; NCT0233266849), atezolizumab (iMATRIX; NCT0254160450), and avelumab (NCT0345182551). These trials were all investigating immunotherapy for recurrent and refractory pediatric tumors. In general, toxicity was reported using NCI CTCAE terminology with no pediatric consensus system for grading and reporting of immune-related adverse effects. It is noted that many cardiovascular metrics, such as blood pressure, are age-dependent and may require a different grading system than that used for adults.

In ADVL1412 (NCT0230445848), nivolumab plus ipilimumab was studied in children and young adults with recurrent/refractory solid tumors (*n* = 53). Under immune-related adverse events, there were two pericardial effusions reported (2/53 incidence) as the only cardiac events [59]. The ongoing KEYNOTE-051 trial examines pembrolizumab (MK-3475) in pediatric patients with solid tumors. Preliminary results of the first phase, reporting on safety and antitumor activity with optimal dosing in advanced pediatric cancer, have been published (*n* = 154) [60]. Cardiotoxicity was uncommon, including only hypertension (*n* = 2, 1%). There was one report of death due to pulmonary edema, but it was not clearly attributed to cardiogenic toxicity, highlighting the need for enhanced reporting of potential cardiovascular effects in clinical trials of immunotherapy in pediatric patients.

The iMATRIX study was a multicenter, open-label international trial of atezolizumab for treatment of patients aged < 30 years with solid tumors or lymphomas [61]. Similar to the KEYNOTE-051 trial, cardiac adverse effects were uncommon and only hypertension was reported (*n* = 1, 1%). Finally, avelumab was studied in a study of children and young adults < 18 years with recurrent/refractory solid tumors [62]. In this study, the only cardiovascular adverse event reported was also blood pressure abnormality, interestingly, in this study both hypotension (*n* = 4) and hypertension (*n* = 2) were reported.

In summary, approvals by the FDA for use of immunotherapy in treating pediatric patients are likely to lead to increased use in this group. Cardiovascular events seem uncommon and mainly manifested as hypertension, but pericardial effusion has been reported and the present data are limited. The pediatric population should be considered specially and would benefit from enhanced reporting of cardiovascular effects with long-term monitoring and discussion on a consensus grading system incorporating age-dependent variables.

### 4.5. Accurate Grading

A generalized consensus concerning a grading scale and proper categorization of cardiovascular symptomology and clinical presentation has yet to be universally utilized. This is likely due to the variety of MACE that may develop. However, the CTCAE has developed a grading of cardiac events based on (1) biomarker outcome, (2) ECG readings, (3) symptoms, and (4) generalized cardiac complications, such as arrhythmia or myocardial ischemia [63]. This grading system provides a guide on properly treating the patient based on the severity of their condition instead of a scale to determine the likelihood an irAE might occur. Grade 1 is defined as close monitoring during treatment in an asymptomatic patient with aberrant cardiac biomarkers and irregular ECG tracings, grade 2 involves minor symptoms, grade 3 is severe symptoms with steroid treatment required, and grade 4 is moderate to severe decompensation of life-threatening situations that demand IV injections of medication or intervention [48]. Utilizing this scale, a grade 1 or 2 is considered mild, whereas a grade 3 or 4 is considered severe [48]. The National Cancer Institute’s Common Toxicity Criteria and the National Comprehensive Cancer Guidelines confirm the grading scale of CTCAE for general cardiovascular complications. The CTCAE allows for proper grading of an ongoing adverse event. However, a more efficient risk assessment system is needed to inform patients and their physicians comprehensively of the next steps in management, particularly the ability to continue treatment or re-challenge.

### 4.6. Prevention

Immunotherapy has revolutionized cancer treatment since its inception, which makes understanding and preventing catastrophic adverse effects of utmost importance. A baseline assessment of cardiac function and analysis of cardiovascular risk factors allows for understanding a patient’s overall cardiac health and proclivity to develop a MACE [54]. Recently, a murine model-based preclinical study analyzed the benefit of TNF-α blockade in targeting early-stage cardiotoxicity based on the PD-1/PD-L1 pathway, exhibiting protective characteristics on the myocardium [13,64]. The study identified that co-administration of TNF-α blockade alongside ICI treatment led to the mitigation of cardiotoxicity, as evidenced by an echocardiogram showing a preserved LVEF of 48.7 ± 3.9 versus 42.1 ± 6.1 for anti-TNF-α/PD1 and anti-PD-1 alone, respectively [65]. Additionally, the benefit of anti-TNF-α compared to previous attempts at CD8+ T cell depletion lies in the idea that using TNF-α blockade does not negate the effects of ICI treatment; this allows for a continuation of immunotherapy while preventing cardiotoxicity, rather than stopping cancer treatment in favor of addressing cardiotoxicity and risking cancer progression [65]. Although the use of TNF-α blockade has not withstood patient-based clinical trials yet, the applications of anti-TNFα remain promising in preventing cardiotoxicity and allowing patients to continue cancer immunotherapy.

Another potential mechanism to prevent cardiac toxicity, specifically the blockade of atherosclerosis progression, may be statin prophylaxis in immunotherapy patients. A clinical study observed a greater than three-fold increase in the aortic plaque volume in melanoma patients treated with ICIs compared to controls, but lower rates of plaque progression and total aortic plaque volume in melanoma patients treated concomitantly with ICIs and a statin compared to immunotherapy alone [23]. Statin therapy becomes especially relevant when observing the effects of PD-1 deficiency leading to significantly increased cellular levels, creating the perfect environment for prevention through statins as a two-edged sword in regulating patient lipid levels and preventing atherosclerosis advancement [23,26].

### 4.7. Management

Managing cardiovascular toxicity in the immunotherapy setting differs from standard symptomatic events in that intravenous high-dose corticosteroids, such as methylprednisolone, are often the first step in treatment [48]. However, a recent review concerning immunotherapy-related cardiovascular complications proposed an eight-point management system to comprehensively evaluate patients, utilizing clinical presentation, ECG, serum cardio biomarkers, TTE, CMR, coronary imaging, and endomyocardial biopsy [49]. Using a multi-point diagnostic reasoning scale allows physicians to understand precisely which type of toxicity they are dealing with, whether PE, myocarditis, ACS, or VTE. Unfortunately, this proposed system recommends that all patients presenting with cardiovascular symptoms be withheld ICI treatment and admitted for telemetry in the case of cardiotoxicity or with regular venous ultrasound or CT imaging in the case of vascular compromise, allowing cancer progression in the meantime [49]. Due to the lack of understanding concerning preventing cardiovascular irAEs, high-dose corticosteroids and cessation of immunotherapy until patient stabilization remain the standard of care [66]. Another proposed option for management of cardiovascular toxicity lies in vitamin D. Based on previous studies investigating the effect of 1.25(OH)_2_ vitamin D_3_ on cardiomyocytes in a murine model of experimental autoimmune myocarditis, markedly reduced apoptosis was observed, as well as decreased caspase expression, a marker of inducible apoptosis within cells [67,68] Given this data, a prospective clinical trial proposal can be to consider vitamin D administration alongside corticosteroid treatment when caring for a patient with ICI-induced cardiovascular toxicity.

## 5. Risk Factors

### 5.1. Clinical Risk Factors

**Figure 2:**
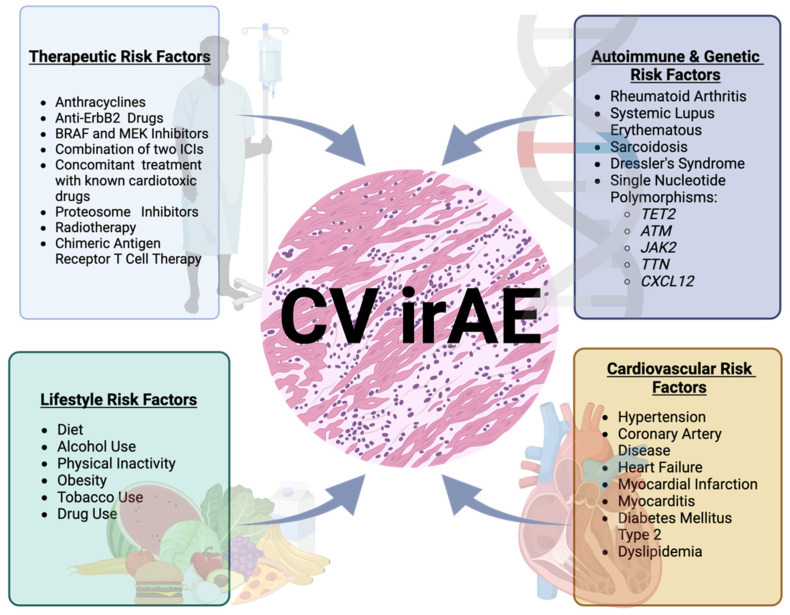
Identified potential risk factors for the development of cardiovascular immune adverse events following or during treatment with immune checkpoint inhibitors categorized by therapeutic risk factors, lifestyle risk factors, autoimmune and genetic risk factors, and independent cardiovascular risk factors. Figure 2 was created using Biorender.

Retrospective studies surveying myocarditis incidence have identified various factors associated with increased risk of cardiovascular adverse events from immunotherapy. These can be divided into patient-specific, tumor-specific, and therapy-specific factors. Patient-specific factors include variables traditionally associated with increased cardiovascular (atherosclerotic) risk, including a history of smoking, alcohol consumption, hypertension, diabetes, advanced age, severe cancer staging at the time of treatment initiation, obesity, or common cardiovascular risk factors such as lack of exercise or family history of cardiovascular disease [40,65]. Tumor-specific factors include tumor genetics and advanced stage at treatment initiation [48]. Figure 2 summarizes risk factors that may increase the chances of a patient developing irAE after or during treatment with ICIs.

Therapy-specific risks may include combination therapy (dual PD-1/PD-L1 and CTLA-4 blockade) and additional irAEs [65]. Additionally, cancer patients are known to have higher rates of thrombosis than the general population, likely due to the hypercoagulable state induced by neoplasia; higher reports of thrombosis are recognized when using platinum-based chemotherapy [69]. Furthermore, in a direct comparison study of patient populations using platinum-based chemotherapy, those receiving immunotherapy, and a combination cohort of platinum-based chemotherapy and immunotherapy, the platinum-based therapy represented a more significant rate of both venous thromboembolism (VTE) and arterial thromboembolism (ATE) than immunotherapy treatment [69]. An important conclusion from these results is that while the risk of VTE or ATE exists with ICIs, the risk is less than the platinum-based chemotherapy agents, such as Cisplatin or Carboplatin, previously utilized as standard therapy. A systematic review with a primary focus on cardiotoxicity related to ICIs with various types of cancer as a risk factor found that melanoma, hematological malignancies, lung cancer, renal cell carcinoma, and urothelial cell carcinoma are at increased risk. The most common type of event noted was hypertension. Hepatocellular carcinoma and malignant pleural mesotheliomas were found to be important risk factors for myocarditis [70].

It has been shown in various studies that an overweight BMI has an increased risk of irAE. A recent study has shown that overweight patients with fewer metabolic comorbid conditions such as diabetes, dyslipidemia, and hypertension were at an increased risk of irAEs [71]. Potential reasons for this may be the inflammatory nature of many of these conditions. Those without comorbid conditions may have a more robust immune response and associated adverse events. This is indirect evidence of these metabolic conditions contributing to cardiovascular adverse events.

An additional patient-specific risk factor implicated in the development of irAEs is a history of preexisting autoimmune disease [72,73,74]; a family history of autoimmune disease may even be enough to predispose an individual to the development of an irAE [75]. Chronic use of cardiotoxic drugs before initiating immunotherapy may also be a risk factor, including anthracyclines, human epidermal growth factor receptor 2 inhibitors, vascular endothelial growth factor, and tyrosine kinase inhibitors [75]. Other irAEs, such as myasthenia gravis or myositis, indicate a higher likelihood of cardiovascular adverse events occurring within a patient, which may indicate the development of an overlapping syndrome utilizing molecular mimicry to assault multiple areas of the body at once [40]. Although numerous correlations throughout studies have been made, research remains ongoing about replicating risk factors to cement guidelines directed at patient eligibility for immunotherapy treatment and administration monitoring strategies.

Differences in response to therapy between sexes may also influence the development of cardiovascular irAEs. Men are more at risk for vascular irAEs, myocarditis, pericarditis, arrhythmia, coronary artery disease, and myocardial infarction than women [75,76]. Age may also influence the risk of irAE development. One study found that older adults had a higher incidence of irAEs; this is thought to be associated with the reduced activity of T-regulatory cells found with aging, which generally provide an anti-inflammatory role by preventing over-activation of the T-cell response [77]. However, other studies have found that an age less than 60 years may be a risk factor for an irAE [75,78].

Venous thromboembolic events such as deep vein thrombosis (DVT), vesicular vein thrombosis VVT, and pulmonary embolism (PE) remain the most prevalent venous thromboembolic immunotherapy-related adverse events. In contrast, acute coronary syndrome (ACS), myocardial infarction (MI), or cerebral ischemia (CI) prove the most common arterial thromboembolic events. Due to the nature of thromboembolic events, the risk factors indicated for cardiovascular toxicity are mirrored as risks for developing thromboembolic events, such as obesity, smoking, progression of cancer, advanced age, and previous history of VTE [56].

Most venous and arterial thrombotic event risk evaluations in chemotherapy treatment are evaluated on the Khorana scale, which provides a risk assessment score predictive of the development of thromboembolic events in cancer patients. The predictive variables include the site of cancer, with 2 points awarded to very high-risk sites and 1 point awarded for moderately high-risk sites, platelet counts of 350 or more, hemoglobin values less than ten, and/or the use of EPO-stimulating agents, white blood cell counts over 11, and BMI of 25 or more, with 1 point awarded for each value over 2.5 [56]. In a study that verified the Khorana score’s validity, VTE rates averaged 0.55, 1.27, and 5 in the low, intermediate, and high-risk cohorts, respectively [56]. Death or disease progression rates were reported as 7%, 18%, and 28% in the low, intermediate, and high-risk cohorts, respectively [56]. Finally, further studies concerning thromboembolic events in ICI-treated cancer patients found a correlation between a higher Khorana score and higher incidence, prevalence, and decreased OS, indicating that the Khorana score may prove a valuable tool in assessing patient VTE or ATE risk during and after ICI-based cancer treatment [55,79]. Although noted in some of the literature, more prospective analysis is necessary to fully synergize the Khorana score with immunotherapy treatment.

Additionally, Garitaonaindia et al. evaluated risk factors for cardiovascular events in patients treated with immunotherapy. The results showed that elder age, history of ischemic heart disease or arrythmia, and reporting other immune-related adverse events are risk factors for developing cardiac events during immunotherapy treatment [80]. Lastly, ICI therapy-associated atherosclerosis has provided additional insights into the potential risk factors for anti-PD-L1 therapy or combined anti-PD-L1 and anti-CTLA4 therapy. Lutgens et al. showed the altered pathophysiology of immune cell composition in coronary atherosclerotic lesions, resulting in an increased CD3/CD68 ratio [80]. Furthermore, it is critical to consider T cell-driven inflammatory responses in human atherosclerotic plaques and elevated CD3/CD68 ratio in plaque instability when evaluating ICI-eligible patients [81].

### 5.2. Identifying Biomarkers in Risk Assessment of ICI-Related Adverse Events

Cancer has a unique capacity to avoid or inhibit the immune system strategically. This is done through the secretion of molecules or recruitment of other cells that stop T cells from effectively operating. Immunotherapy aims to treat cancer by overcoming these defenses. Meanwhile, biomarker identification allows researchers to measure and predict specific aspects of an individual’s health. In immunotherapy, biomarkers can provide information toward personalized cancer therapy. Specific biomarkers are critical for understanding genetic makeup, cancer behavior, and immune system interactions. Recent advances have shown that saliva, breath, urine, and stool can also provide important biomarker information. Several biomarkers such as PD-1/PD-L1, CTLA-4, and combinations have offered promising results. Specifically, PD-1/PD-L1 expression was evaluated by the CRI-SU2C Dream Team, which revealed the association between PD-L1 expression and responses to PD-1/PD-L1 checkpoint immunotherapy in patients with various types of cancers [82].

Biomarker-driven cancer therapies further allow clinicians to identify the unique characteristics of an individual’s cancer and provide targeted and personalized treatment. Additionally, biomarkers will provide information on potential immune adverse events during or after immunotherapy. Specifically, biomarker information could predict adverse patient effects and offer the opportunity to provide prophylactic measures. Mice studies conducted on TIM-3 negative subjects resulted in decreased STAT-3 expression. This reduced IL-10, EBI3, Granzyme B, Perforin, IL-1Rα, and CCR6 expression. The overall effect is decreased inhibition of Th1 and Th17 cells [83]. This study implies that human subjects lacking TIM-3 could be at significant risk of developing irAE in cardiac cells due to the consistent T-cell-mediated response in the heart. An overall decreased gene expression of these biomarkers can be a potential window into what is causing irAE in cardiac tissues. A retrospective analysis of cardiovascular events in patients with lung cancer receiving ICIs was done to help provide useful biomarkers. They found that the neutrophil-to-lymphocyte ratio and C reactive protein were higher in patients with cardiovascular events. The authors concluded that these would be useful biomarkers in screening for irAEs in those treated with ICIs. The results showed that NLR had a larger impact than CRP, but CRP was elevated at the time of the event [84]. Another preclinical study about cardiac complications showed increased expression of NF-kB, systemic SDF-1, IL-1β, IL-6 levels, and myocardial NLRP3, MyD88, and damage-associated molecular patterns (DAMPs) [85]. These subjects were exposed to PD-1 and CTLA-4-blocking agents [85]. This resulted in a cytokine storm in myocardial tissue. As outlined in previous mice models, subjects with decreased PD-1 or CTLA-4 will be at increased risk of cardiac cells. Increased levels of these outlined biomarkers may not be good candidates for immunotherapy due to the risk of cardiovascular events. Other considerations will have to be made, including the patient’s overall risk of irAE and general cardiovascular risk factors, before starting ICI therapy.

A recent immune checkpoint inhibitor discovered is LAG3, which is expressed on CD4(+) and CD8(+) cells and T regs. With the role of this ICI in T cell exhaustion in cancer, it will be important to consider this as a mechanism in cardiovascular events in those without LAG-3, like the PD1(-/-) and CTLA-4-negative mice [86]. PD-L1, PD-1, CTLA-4, and LAG-3 deficiency in mice has been outlined in this paper as a potential mechanism of the cause of cardiovascular events. Another important factor to consider is that interferon-gamma secreted by T cells helps upregulate the high number of PD-1 cells in the heart [87]. This again shows the critical role of PD-1 in the heart in preventing irAEs. T cells localized in the heart that are not secreting large amounts of IFN-g may leave individuals at risk for such events.

Another risk factor for irAEs was found in those treated with the cytokine IL-2. This included tachycardia, edema, decreased left ventricular stroke work index, angina, ischemic changes, hypotension, and arrhythmias, primarily supraventricular. This paper states that those with underlying cardiorespiratory disease may be at increased risk of such events [88]. While our primary focus is on ICI irAEs, these findings indicate that those with higher IL-2 levels may have an increased risk of irAEs in cardiac tissues. Another potential biomarker is plasma and cardiac Galectin-3 (Gal-3). This molecule is pro-inflammatory. Biopsies in the endocardium for those with cardiomyopathy and myocarditis showed that it was correlated with inflammatory infiltrate in the heart. They concluded that Gal-3 should be considered as a possible marker for cardiac inflammation and fibrosis [89]. For our purposes, an elevated level of this biomarker could be a future predictor of cardiac irAE in those receiving immunotherapy. Further study will need to include subjects with or without elevation in this biomarker before receiving the immunotherapy to determine if this will have predictive value in immunotherapy management.

### 5.3. Genetics as Risk Factors

Identification and analysis of genetic composition have emerged as a potential tool to guide personalized immunotherapies. Much of the literature aims to discuss the shared genetic risk factors between cancer and cardiovascular disease. However, potential connections between specific genetic markers and cardiovascular irAEs remain tentative and bear potential for further research. One clear genetic risk factor for the development of ICI irAEs are polymorphisms of CTLA-4, PD-1, or PDL-1 [90]. Turk and Kunej discussed the shared genetic risk factors for cardiovascular disease. A string diagram was created to network identified loci (genes and SNP) and their association with cancer types and cardiovascular diseases. The network identified JAK2, TTN, TET2, and ATM with 16, 12, 8, and 6 edges, respectively [91]. These preliminary genetic identifications could be further evaluated in immunotherapy patients and linked to cardiotoxicity-related adverse events.

One study by Udagawa et al. aimed to identify genetic variants in the Japanese population associated with nivolumab-provoked irAEs, finding possible associations with 90 single nucleotide polymorphisms (SNPs). These included a SNP found upstream of the gene for Amyloid Beta Precursor protein, which is downregulated in Crohn’s. Also potentially implicated was a SNP downstream of the gene for the TNF superfamily member 14, a ligand for the lymphotoxin β receptor, which stimulates T-cell differentiation and growth; upregulation of this gene is associated with a variety of T-cell-mediated diseases, including various autoimmune diseases. An additional SNP potentially associated with irAEs was located in the gene for an adaptor protein regulating the binding of a transcription factor in the ETS transcription factor family, associated with immune cell development [92]. Several other studies have similarly found SNPs associated with increased risk, decreased risk, or increased severity of irAE development [75]. Although none of these were specifically associated with cardiovascular irAEs, the development of irAEs is multifactorial and may involve several genetic factors. These findings imply that genetic variants associated with cardiovascular irAEs may be uncovered with further research. More general biomarkers related to heart failure and increased all-cause mortality risk include stromal cell-derived factor 1α (SDF-1) which CXCL12 encodes. This locus is associated with coronary artery disease [93]. The risk of cardiovascular heart failure is an important biomarker that is associated with older age, lower HDL cholesterol, and cigarette smoking. This points to these factors as potential risk factors for those already at risk for cardiovascular events before receiving immunotherapy.

The shared biology between cancer and cardiovascular disease has also been linked to diet. Specifically, genetic mutations in the folate metabolism pathway, in conjugation with minimal folate intake, are associated with an increased risk of cardiovascular disease and colorectal cancer. Lastly, the genetic components of race predispose individuals toward nonmodifiable risk factors. Single nucleotide polymorphisms in linkage disequilibrium often demonstrated higher allele frequencies in certain populations, possibly linked to cardiovascular diseases [93,94].

## 6. Conclusions

Immunotherapy is a promising and rapidly emerging novel treatment option for various cancer subtypes. As with any medical intervention, immunotherapy does not come without risks for notable clinical complications. Cardiovascular toxicity following the introduction of immunotherapy is an increasingly documented finding that can introduce novel cardiac complications in cancer patients or worsen a patient’s baseline cardiac functionality. Given the rapid expansion of immunotherapy in both research discovery and patient care, clinicians need to understand potential mechanisms behind toxicity, the risk factors related to adverse events, and strategies to prevent toxicity before it occurs. We previously documented multiple risk factors that correlate with an increased risk of immune-related adverse event irAEs, major cardiac adverse events, and AVE. These risk factors included pharmacological history, treatment-related factors, autoimmune conditions, cardiovascular factors, tumor-related factors, social factors, genetic factors, and immune-related factors. The vast majority of these risk factors can be uncovered by a well-executed history and physical exam by an astute clinician. Therefore, medical education regarding the warning signs related to the risk factors of immunotoxicity is crucial.

A current limitation to our findings is that while many risk factors for irAE’s have been identified, they have yet to be stratified into an effective clinical screening tool. In the future, it may be beneficial to generate national guidelines or a numerical scale/score that utilizes these risk factors to assess a cancer patient’s overall probability of developing irAEs following the introduction of immunotherapy. This would potentially serve as a screening tool and overall preventative measure to identify patients at high risk for irAEs before treatment is initiated. Essentially, this could be used to determine if a patient qualifies as a good candidate for immunotherapy. Furthermore, investigation into genetic signatures and biomarkers may provide further insight into individuals highly predisposed to irAEs.

Once immunotherapy treatment is approved, many actions should be taken to assess a patient’s overall baseline cardiovascular function. This includes obtaining a baseline EKG and echocardiogram. These tests allow a clinician to assess for any baseline electrical or ejection fraction abnormalities and offer a valuable comparison tool to any subsequent testing throughout an immunotherapy treatment. Upon any initial incidence of acute symptoms of MACE, a physician will then be able to monitor the progression/severity of ongoing toxicity effectively. We also described future potential for patients to take prophylactic medication to prevent irAEs during a course of immunotherapy. This prophylaxis may feature supplemental anti-inflammatory medications to be taken alongside immunotherapy. In particular, anti-TNFα therapy has shown promise in animal studies to reduce the odds of irAE manifestation in the context of immunotherapy. These promising findings suggest that clinical trials may warrant investigation.

Overall, the emerging field of immunotherapy has the potential to offer immense benefits to a wide range of cancer patients. Once treatment is initiated, it is vital for clinicians to take the necessary steps to anticipate, prevent, recognize, and assess cardiotoxicity-related side effects. These actions will ultimately lead to significant improvement in the quality of patient care. Additionally, further research and investigation is necessary to promote optimal and safe ways to administer immunotherapy.

## Figures and Tables

**Table 1:** Overview of ICI-related cardiovascular toxicity.

ICI-Related Cardiotoxicity	Presentation	Biomarkers/Laboratory Studies	Labs and Imaging	Management	Common ICI Drugs Responsible
Myocarditis [34,35,36,37,38]	Presentation ranges from symptomatic to cardiac decompensation May present with HF, pulmonary edema, cardiogenic shock, arrhythmias or multiorgan failure.	↑ Troponins in in >90% patients. Troponin T >1.5 ng/mL predictive of MACE. ↑ creatinine kinase MB Troponin and CK-MB used at baseline to identify high-risk patients. ↑ BNP and NT pro-BNP in heart failure patients (though it is chronically elevated in patients with cancer). NT pro-BNP trending level predict response to therapy.	Electrocardiogram First test performed Findings often nonspecific: conduction abnormalities like sinus arrhythmia, atrial or ventricular arrhythmias, QRS interval prolongation, and QT prolongation. Can help diagnose new onset conduction blocks Echocardiography TTE -decrease in LVEF LVEF reduction >10% to below the lower limit of normal is diagnostic GLS with 2 is diagnostic and prognostic GLS > 15% reduction from baseline is diagnostic MUGA scan is reproducible LVEF decrease > 10% Cardiac MRI Preferred test, superior to echocardiography; highly sensitive and specific used for LV function assessment in borderline cases T2-weighted imaging visualize myocardial inflammation and edema CMR differentiates myocarditis from other cardiac dysfunction. Endomyocardial biopsy Most specific diagnostic test for ICI myocarditis. confirm the diagnosis of myocarditis Is invasive so, limited clinical value but can be used if the diagnosis is uncertain Biopsy shows -inflammatory infiltrates with lymphocytes and macrophage, myocardial fibrosis. presence of cell-specific markers CD3, CD68, or human leukocyte antigens	On suspicion of myocarditis, Stop ICI therapy, proceed to grading per ASCO guidelines G1-mildly abnormal screening test with no symptoms G2 minimally symptomatic G3 symptomatic G4 unstable G-1 G2 work up to confirm ICI-related myocarditis; if confirmed, start treatment for myocarditis. Methylprednisolone 500–1000 mg daily until clinically stable, Followed by oral prednisone 1 mg/kg once daily, and then slow taper of steroid over 4 to 6 weeks. After cardiac function returns to normal, consider other immunosuppressive therapies In steroid non-responders -plasmapheresis, iv- immunoglobulins, anti-thymocyte globulin (ATG), mycophenolate mofetil, tacrolimus and infliximab Other adjuvant drugs-diuretics, β-blockers, ACEI drugs, antiarrhythmic drugs Consider permanent discontinuation of ICI for myocarditis grades 2 to 4 [34,39,40].	Pembrolizumab Nivolumab Atezolizumab Avelumab Durvalumab Ipilimumab
Pericardial disease [37,38,41,42]	2nd most common cardiotoxicity with ICI therapy can present as isolated pericarditis (chest pain and dyspnea, may rapidly progress to respiratory failure) or with myocarditis or as pericardial effusion or tamponade.	↑ Troponin levels if present with myocarditis. pericardial fluid cytology: lymphocyte-rich infiltrate and the absence of malignant cells help differentiate from cancer-associated pericardial effusion	Echocardiograms and chest CT can effectively detect pericardial diseases Electrocardiogram ST-T changes, low voltage complexes Echocardiography for evaluating pericardial effusion TTE: constrictive-effusion, tamponade CMR assessing myocardial involvement.	Stop ICI therapy Methylprednisolone 500–1000 mg daily until clinically stable, followed by oral prednisone 1 mg/kg once daily, and wean as tolerated. Colchicine and NSAIDs as adjunctive treatment Steroid-refractory pericarditis, consider mycophenolate mofetil, infliximab, or anti-thymocyte globulin. Pericardiocentesis if cardiac tamponade is present	Pembrolizumab Nivolumab Atezolizumab Avelumab Durvalumab Ipilimumab
Cardiomyopathy [42,43,44,45]	Heart failure: dyspnea, volume overload, edema, Takotsubo cardiomyopathy acute reversible LV systolic dysfunction, mimicking acute coronary syndrome in the absence of obstructive coronary artery disease	↑ pro-BNP levels	Electrocardiogram [36] sinus tachycardia, QT prolongation, ST-segment elevation, T inversion Echocardiography TTE -moderate to severely depressed LV function, ↓ LVEF, ↑ PA pressure, apical akinesia, medial and basal segments akinesia, apical ballooning pattern, and reduced left ventricular GLS. Coronary angiography to rule out acute coronary syndrome CMR to exclude myocarditis. It may or may not show LGE consistent with concomitant myopericarditis Endomyocardial biopsy myocardial fibrosis	Cessation of ICI therapy heart failure therapy; Diuretics Corticosteroids Beta blockers in Takotsubo cardiomyopathy	ipilimumab nivolumab atezolizumab
Cardiac conduction disease [42]	Usually associated with myocarditis Variable presentation- isolated atrial or ventricular extra systole supraventricular arrhythmias, atrioventricular block	NA	Electrocardiogram corresponds to the type of conduction defect	Stop ICI therapy Management of arrhythmias per ACC guidelines	Pembrolizumab Nivolumab Atezolizumab Avelumab Ipilimumab
Vasculitis [42]	Temporal arteritis: visual impairment Polymyalgia rheumatica: malaise Cerebral vasculitis	High ESR and CRP	NA	Corticosteroids	Pembrolizumab Nivolumab Ipilimumab
Coronary artery disease [42,46,47]	Atherosclerotic plaque progression, myocardial infarction, or stroke		Electrocardiogram ST elevations and new onset Q waves. Coronary computed tomography Identify acute coronary syndrome. total plaque volume progression.		nivolumab, pembrolizumab, atezolizumab, avelumab, and durvalumab ipilimumab, pembrolizumab

ICI—immune checkpoint inhibitors, ↑—elevated, ↓—reduced, BNP—brain natriuretic peptide, NT—proBNP–N-terminal pro–B-type natriuretic peptide, Trop T—troponin T, MACE—major adverse cardiac event, TTE—trans thoracic echocardiography, GLS—global longitudinal strain, ASCO—American Society of Clinical Oncology, MUGA—multigated acquisition scan, LV—left ventricle, LVEF—left ventricle ejection fraction, MRI—magnetic resonance imaging, EKG—electrocardiogram, CMR—cardiac magnetic resonance imaging, NSAIDS—nonsteroidal anti-inflammatory drugs, NA—not applicable PA—pulmonary artery, ACC—American College of Cardiology.

## Data Availability

No new data were created or analyzed in this study. Data sharing is not applicable to this article.

## Abbreviations

ACS	Acute Coronary Syndrome
ACT	Adoptive Cell Therapy
APC	Antigen-Presenting Cell
ATE	Arterial Thromboembolic Event
AVE	Adverse Vascular Event
BMI	Body Mass Index
BNP	Brain Natriuretic Peptide
CAR	Chimeric Antigen Receptor
CI	Cerebral Ischemia
CMR	Cardiac Magnetic Resonance
CR	Complete Remission
CRP	C-Reactive Protein
CTCAE	Common Terminology Criteria for Adverse Events
DC	Dendritic Cell
DVT	Deep Vein Thrombosis
ECG	Electrocardiogram
EPO	Erythropoietin
FDA	Food and Drug Administration
GAL-3	Galectin-3
GLS	Global Longitudinal Strain
GM-CSF	Granulocyte-Macrophage Colony Stimulating Factor
ICI	Immune Checkpoint Inhibitor
ICAM-1	Intracellular Adhesion Molecule-1
IFN-g	Interferon gamma
IL	Interleukin
irAE	Immune-Related Adverse Event
ITAM	Immunoreceptor Tyrosine-based Activation Motif
ITIM	Immunoreceptor Tyrosine-based Inhibition Motif
ITSM	Immunoreceptor Tyrosine-based Switch Motif
LDH	Lactate Dehydrogenase
LDL	Low-Density Lipoprotein
LN	Lymph Node
LV	Left Ventricular
LVEF	Left Ventricular Ejection Fraction
mAb	monoclonal Antibody
MACE	Major Adverse Cardiac Events
MDSC	Myeloid-Derived Suppressor Cell
MI	Myocardial Infarction
MUGA	Multigated Radionuclide Angiography
NF-κB	Nuclear Factor Kappa B
NK	Natural Killer
NLR	Neutrophil-to-Lymphocyte Ratio
NO	Nitric Oxide
OS	Overall Survival
ORR	Overall Response Rate
PE	Pulmonary Embolism
ROS	Reactive Oxygen Species
SDF-1	Stromal cell-derived factor 1 alpha
SNP	Single Nucleotide Polymorphism
TCR	T Cell Receptor
TGF-b	TGF-beta
Th	T helper
TIL	Tumor-Infiltrating Lymphocyte
TME	Tumor Microenvironment
TNF-a	TNF-alpha
Treg	T Regulatory Cell
VCAM-1	Vascular Adhesion Molecule-1
VTE	Venous Thromboembolic Event
VVT	Vesicular Vein Thrombosis

## References

[1] Guerder S., Flavell R.A. (1995). T-cell activation. Two for T. Curr. Biol..

[2] Hunter M.C., Teijeira A., Halin C. (2016). T Cell Trafficking through Lymphatic Vessels. Front. Immunol..

[3] Gajewski T.F., Schreiber H., Fu Y.X. (2013). Innate and adaptive immune cells in the tumor microenvironment. Nat. Immunol..

[4] Galluzzi L., Vacchelli E., Bravo-San Pedro J.M., Buque A., Senovilla L., Baracco E.E., Bloy N., Castoldi F., Abastado J.P., Agostinis P. (2014). Classification of current anticancer immunotherapies. Oncotarget.

[5] Baxter D. (2014). Active and passive immunization for cancer. Hum. Vaccin. Immunother..

[6] Topalian S.L., Drake C.G., Pardoll D.M. (2015). Immune checkpoint blockade: A common denominator approach to cancer therapy. Cancer Cell.

[7] Hodi F.S., O’Day S.J., McDermott D.F., Weber R.W., Sosman J.A., Haanen J.B., Gonzalez R., Robert C., Schadendorf D., Hassel J.C. (2010). Improved survival with ipilimumab in patients with metastatic melanoma. N. Engl. J. Med..

[8] Robert C., Thomas L., Bondarenko I., O’Day S., Weber J., Garbe C., Lebbe C., Baurain J.F., Testori A., Grob J.J. (2011). Ipilimumab plus dacarbazine for previously untreated metastatic melanoma. N. Engl. J. Med..

[9] Robert C., Long G.V., Brady B., Dutriaux C., Maio M., Mortier L., Hassel J.C., Rutkowski P., McNeil C., Kalinka-Warzocha E. (2015). Nivolumab in previously untreated melanoma without BRAF mutation. N. Engl. J. Med..

[10] Larkin J., Chiarion-Sileni V., Gonzalez R., Grob J.J., Rutkowski P., Lao C.D., Cowey C.L., Schadendorf D., Wagstaff J., Dummer R. (2019). Five-Year Survival with Combined Nivolumab and Ipilimumab in Advanced Melanoma. N. Engl. J. Med..

[11] Shoushtari A.N., Friedman C.F., Navid-Azarbaijani P., Postow M.A., Callahan M.K., Momtaz P., Panageas K.S., Wolchok J.D., Chapman P.B. (2018). Measuring Toxic Effects and Time to Treatment Failure for Nivolumab Plus Ipilimumab in Melanoma. JAMA Oncol..

[12] Tivol E.A., Borriello F., Schweitzer A.N., Lynch W.P., Bluestone J.A., Sharpe A.H. (1995). Loss of CTLA-4 leads to massive lymphoproliferation and fatal multiorgan tissue destruction, revealing a critical negative regulatory role of CTLA-4. Immunity.

[13] Okazaki T., Tanaka Y., Nishio R., Mitsuiye T., Mizoguchi A., Wang J., Ishida M., Hiai H., Matsumori A., Minato N. (2003). Autoantibodies against cardiac troponin I are responsible for dilated cardiomyopathy in PD-1-deficient mice. Nat. Med..

[14] Keir M.E., Liang S.C., Guleria I., Latchman Y.E., Qipo A., Albacker L.A., Koulmanda M., Freeman G.J., Sayegh M.H., Sharpe A.H. (2006). Tissue expression of PD-L1 mediates peripheral T cell tolerance. J. Exp. Med..

[15] Johnson D.B., Balko J.M., Compton M.L., Chalkias S., Gorham J., Xu Y., Hicks M., Puzanov I., Alexander M.R., Bloomer T.L. (2016). Fulminant Myocarditis with Combination Immune Checkpoint Blockade. N. Engl. J. Med..

[16] Grabie N., Gotsman I., DaCosta R., Pang H., Stavrakis G., Butte M.J., Keir M.E., Freeman G.J., Sharpe A.H., Lichtman A.H. (2007). Endothelial programmed death-1 ligand 1 (PD-L1) regulates CD8+ T-cell mediated injury in the heart. Circulation.

[17] Nishimura H., Okazaki T., Tanaka Y., Nakatani K., Hara M., Matsumori A., Sasayama S., Mizoguchi A., Hiai H., Minato N. (2001). Autoimmune dilated cardiomyopathy in PD-1 receptor-deficient mice. Science.

[18] Ceschi A., Noseda R., Palin K., Verhamme K. (2020). Immune Checkpoint Inhibitor-Related Cytokine Release Syndrome: Analysis of WHO Global Pharmacovigilance Database. Front. Pharmacol..

[19] Mohan M.L., Vasudevan N.T., Prasad S.V.N. (2017). Pro-inflammatory cytokines mediate GPCR dysfunction. J. Cardiovasc. Pharmacol..

[20] Bar J., Markel G., Gottfried T., Percik R., Leibowitz-Amit R., Berger R., Golan T., Daher S., Taliansky A., Dudnik E. (2019). Acute vascular events as a possibly related adverse event of immunotherapy: A single-institute retrospective study. Eur. J. Cancer.

[21] Fan J., Watanabe T. (2003). Inflammatory reactions in the pathogenesis of atherosclerosis. J. Atheroscler. Thromb..

[22] Hansson G.K. (2001). Immune mechanisms in atherosclerosis. Arterioscler. Thromb. Vasc. Biol..

[23] Drobni Z.D., Alvi R.M., Taron J., Zafar A., Murphy S.P., Rambarat P.K., Mosarla R.C., Lee C., Zlotoff D.A., Raghu V.K. (2020). Association Between Immune Checkpoint Inhibitors with Cardiovascular Events and Atherosclerotic Plaque. Circulation.

[24] Kondo S., Sato N., Aso K. (1991). The level of urinary epidermal growth factor is not influenced by the extent of psoriatic lesions. Arch. Dermatol. Res..

[25] Fernandez D.M., Rahman A.H., Fernandez N.F., Chudnovskiy A., Amir E.D., Amadori L., Khan N.S., Wong C.K., Shamailova R., Hill C.A. (2019). Single-cell immune landscape of human atherosclerotic plaques. Nat. Med..

[26] Strauss L., Mahmoud M.A.A., Weaver J.D., Tijaro-Ovalle N.M., Christofides A., Wang Q., Pal R., Yuan M., Asara J., Patsoukis N. (2020). Targeted deletion of PD-1 in myeloid cells induces antitumor immunity. Sci. Immunol..

[27] Poels K., van Leent M.M.T., Reiche M.E., Kusters P.J.H., Huveneers S., de Winther M.P.J., Mulder W.J.M., Lutgens E., Seijkens T.T.P. (2020). Antibody-Mediated Inhibition of CTLA4 Aggravates Atherosclerotic Plaque Inflammation and Progression in Hyperlipidemic Mice. Cells.

[28] Kyaw T., Winship A., Tay C., Kanellakis P., Hosseini H., Cao A., Li P., Tipping P., Bobik A., Toh B.H. (2013). Cytotoxic and proinflammatory CD8+ T lymphocytes promote development of vulnerable atherosclerotic plaques in apoE-deficient mice. Circulation.

[29] Ramos-Casals M., Brahmer J.R., Callahan M.K., Flores-Chavez A., Keegan N., Khamashta M.A., Lambotte O., Mariette X., Prat A., Suarez-Almazor M.E. (2020). Immune-related adverse events of checkpoint inhibitors. Nat. Rev. Dis. Primers.

[30] Chhabra N., Kennedy J. (2021). A Review of Cancer Immunotherapy Toxicity: Immune Checkpoint Inhibitors. J. Med. Toxicol..

[31] Robert C. (2020). A decade of immune-checkpoint inhibitors in cancer therapy. Nat. Commun..

[32] Antonia S.J., Villegas A., Daniel D., Vicente D., Murakami S., Hui R., Kurata T., Chiappori A., Lee K.H., de Wit M. (2018). Overall Survival with Durvalumab after Chemoradiotherapy in Stage III NSCLC. N. Engl. J. Med..

[33] Tajiri K., Sekine I. (2022). Atherosclerotic cardiovascular events associated with immune checkpoint inhibitors in cancer patients. Jpn. J. Clin. Oncol..

[34] Palaskas N., Lopez-Mattei J., Durand J.B., Iliescu C., Deswal A. (2020). Immune Checkpoint Inhibitor Myocarditis: Pathophysiological Characteristics, Diagnosis, and Treatment. J. Am. Heart Assoc..

[35] Lehmann L.H., Cautela J., Palaskas N., Baik A.H., Meijers W.C., Allenbach Y., Alexandre J., Rassaf T., Müller O.J., Aras M. (2021). Clinical Strategy for the Diagnosis and Treatment of Immune Checkpoint Inhibitor–Associated Myocarditis: A Narrative Review. JAMA Cardiol..

[36] Power J.R., Alexandre J., Choudhary A., Ozbay B., Hayek S., Asnani A., Tamura Y., Aras M., Cautela J., Thuny F. (2021). Electrocardiographic Manifestations of Immune Checkpoint Inhibitor Myocarditis. Circulation.

[37] Altan M., Toki M.I., Gettinger S.N., Carvajal-Hausdorf D.E., Zugazagoitia J., Sinard J.H., Herbst R.S., Rimm D.L. (2019). Immune Checkpoint Inhibitor-Associated Pericarditis. J. Thorac. Oncol..

[38] Zhou Y.W., Zhu Y.J., Wang M.N., Xie Y., Chen C.Y., Zhang T., Xia F., Ding Z.Y., Liu J.Y. (2019). Immune Checkpoint Inhibitor-Associated Cardiotoxicity: Current Understanding on Its Mechanism, Diagnosis and Management. Front. Pharmacol..

[39] Wu Y., Xu Y., Xu L. (2023). Drug therapy for myocarditis induced by immune checkpoint inhibitors. Front. Pharmacol..

[40] Patel R.P., Parikh R., Gunturu K.S., Tariq R.Z., Dani S.S., Ganatra S., Nohria A. (2021). Cardiotoxicity of Immune Checkpoint Inhibitors. Curr. Oncol. Rep..

[41] Gong J., Drobni Z.D., Zafar A., Quinaglia T., Hartmann S., Gilman H.K., Raghu V.K., Gongora C., Sise M.E., Alvi R.M. (2021). Pericardial disease in patients treated with immune checkpoint inhibitors. J. ImmunoTherapy Cancer.

[42] Mocan-Hognogi D.L., Trancǎ S., Farcaş A.D., Mocan-Hognogi R.F., Pârvu A.V., Bojan A.S. (2021). Immune Checkpoint Inhibitors and the Heart. Front. Cardiovasc. Med..

[43] Brumberger Z.L., Branch M.E., Klein M.W., Seals A., Shapiro M.D., Vasu S. (2022). Cardiotoxicity risk factors with immune checkpoint inhibitors. Cardio-Oncol..

[44] Geisler B.P., Raad R.A., Esaian D., Sharon E., Schwartz D.R. (2015). Apical ballooning and cardiomyopathy in a melanoma patient treated with ipilimumab: A case of takotsubo-like syndrome. J. Immunother. Cancer.

[45] Trontzas I.P., Vathiotis I.A., Kyriakoulis K.G., Sofianidi A., Spyropoulou Z., Charpidou A., Kotteas E.A., Syrigos K.N. (2023). Takotsubo Cardiomyopathy in Cancer Patients Treated with Immune Checkpoint Inhibitors: A Systematic Review and Meta-Summary of Included Cases. Cancers.

[46] Suero-Abreu G.A., Zanni M.V., Neilan T.G. (2022). Atherosclerosis With Immune Checkpoint Inhibitor Therapy: Evidence, Diagnosis, and Management: JACC: CardioOncology State-of-the-Art Review. JACC CardioOncol.

[47] Inno A., Chiampan A., Lanzoni L., Verzè M., Molon G., Gori S. (2021). Immune Checkpoint Inhibitors and Atherosclerotic Vascular Events in Cancer Patients. Front. Cardiovasc. Med..

[48] Lei Y., Zheng X., Huang Q., Li X., Qiu M., Liu M. (2022). Intrinsic Differences in Immune Checkpoint Inhibitor-Induced Myocarditis: A Retrospective Analysis of Real World Data. Front. Pharmacol..

[49] Thuny F., Naidoo J., Neilan T.G. (2022). Cardiovascular complications of immune checkpoint inhibitors for cancer. Eur. Heart J..

[50] Blancas I., Martin-Perez F.J., Garrido J.M., Rodriguez-Serrano F. (2020). NT-proBNP as predictor factor of cardiotoxicity during trastuzumab treatment in breast cancer patients. Breast.

[51] Kittiwarawut A., Vorasettakarnkij Y., Tanasanvimon S., Manasnayakorn S., Sriuranpong V. (2013). Serum NT-proBNP in the early detection of doxorubicin-induced cardiac dysfunction. Asia Pac. J. Clin. Oncol..

[52] Sawaya H., Sebag I.A., Plana J.C., Januzzi J.L., Ky B., Tan T.C., Cohen V., Banchs J., Carver J.R., Wiegers S.E. (2012). Assessment of echocardiography and biomarkers for the extended prediction of cardiotoxicity in patients treated with anthracyclines, taxanes, and trastuzumab. Circ. Cardiovasc. Imaging.

[53] Plana J.C., Galderisi M., Barac A., Ewer M.S., Ky B., Scherrer-Crosbie M., Ganame J., Sebag I.A., Agler D.A., Badano L.P. (2014). Expert consensus for multimodality imaging evaluation of adult patients during and after cancer therapy: A report from the American Society of Echocardiography and the European Association of Cardiovascular Imaging. Eur. Heart J. Cardiovasc. Imaging.

[54] Esposito R., Fedele T., Orefice S., Cuomo V., Prastaro M., Canonico M.E., Ilardi F., De Stefano F., Fiorillo L., Santoro C. (2021). An Emergent Form of Cardiotoxicity: Acute Myocarditis Induced by Immune Checkpoint Inhibitors. Biomolecules.

[55] Sheng I.Y., Gupta S., Reddy C.A., Angelini D., Funchain P., Sussman T.A., Sleiman J., Ornstein M.C., McCrae K., Khorana A.A. (2021). Thromboembolism in Patients with Metastatic Renal Cell Carcinoma Treated with Immunotherapy. Target. Oncol..

[56] Khorana A.A. (2010). Venous thromboembolism and prognosis in cancer. Thromb. Res..

[57] Luke J.J., Rutkowski P., Queirolo P., Del Vecchio M., Mackiewicz J., Chiarion-Sileni V., de la Cruz Merino L., Khattak M.A., Schadendorf D., Long G.V. (2022). Pembrolizumab versus placebo as adjuvant therapy in completely resected stage IIB or IIC melanoma (KEYNOTE-716): A randomised, double-blind, phase 3 trial. Lancet.

[58] Chen A.P., Sharon E., O’Sullivan-Coyne G., Moore N., Foster J.C., Hu J.S., Van Tine B.A., Conley A.P., Read W.L., Riedel R.F. (2023). Atezolizumab for Advanced Alveolar Soft Part Sarcoma. N. Engl. J. Med..

[59] Davis K.L., Fox E., Isikwei E., Reid J.M., Liu X., Minard C.G., Voss S., Berg S.L., Weigel B.J., Mackall C.L. (2022). A Phase I/II Trial of Nivolumab plus Ipilimumab in Children and Young Adults with Relapsed/Refractory Solid Tumors: A Children’s Oncology Group Study ADVL1412. Clin. Cancer Res..

[60] Geoerger B., Kang H.J., Yalon-Oren M., Marshall L.V., Vezina C., Pappo A., Laetsch T.W., Petrilli A.S., Ebinger M., Toporski J. (2020). Pembrolizumab in paediatric patients with advanced melanoma or a PD-L1-positive, advanced, relapsed, or refractory solid tumour or lymphoma (KEYNOTE-051): Interim analysis of an open-label, single-arm, phase 1-2 trial. Lancet Oncol..

[61] Geoerger B., Zwaan C.M., Marshall L.V., Michon J., Bourdeaut F., Casanova M., Corradini N., Rossato G., Farid-Kapadia M., Shemesh C.S. (2020). Atezolizumab for children and young adults with previously treated solid tumours, non-Hodgkin lymphoma, and Hodgkin lymphoma (iMATRIX): A multicentre phase 1-2 study. Lancet Oncol..

[62] Loeb D.M., Lee J.W., Morgenstern D.A., Samson Y., Uyttebroeck A., Lyu C.J., Van Damme A., Nysom K., Macy M.E., Zorzi A.P. (2022). Avelumab in paediatric patients with refractory or relapsed solid tumours: Dose-escalation results from an open-label, single-arm, phase 1/2 trial. Cancer Immunol. Immunother..

[63] Brahmer J.R., Lacchetti C., Schneider B.J., Atkins M.B., Brassil K.J., Caterino J.M., Chau I., Ernstoff M.S., Gardner J.M., Ginex P. (2018). Management of Immune-Related Adverse Events in Patients Treated with Immune Checkpoint Inhibitor Therapy: American Society of Clinical Oncology Clinical Practice Guideline. J. Clin. Oncol..

[64] Tarrio M.L., Grabie N., Bu D.X., Sharpe A.H., Lichtman A.H. (2012). PD-1 protects against inflammation and myocyte damage in T cell-mediated myocarditis. J. Immunol..

[65] Michel L., Helfrich I., Hendgen-Cotta U.B., Mincu R.I., Korste S., Mrotzek S.M., Spomer A., Odersky A., Rischpler C., Herrmann K. (2022). Targeting early stages of cardiotoxicity from anti-PD1 immune checkpoint inhibitor therapy. Eur. Heart J..

[66] Zhang L., Zlotoff D.A., Awadalla M., Mahmood S.S., Nohria A., Hassan M.Z.O., Thuny F., Zubiri L., Chen C.L., Sullivan R.J. (2020). Major Adverse Cardiovascular Events and the Timing and Dose of Corticosteroids in Immune Checkpoint Inhibitor-Associated Myocarditis. Circulation.

[67] Hu F., Yan L., Lu S., Ma W., Wang Y., Wei Y., Yan X., Zhao X., Chen Z., Wang Z. (2016). Effects of 1, 25-Dihydroxyvitamin D3 on Experimental Autoimmune Myocarditis in Mice. Cell Physiol. Biochem..

[68] Bhalla A.K., Amento E.P., Clemens T.L., Holick M.F., Krane S.M. (1983). Specific high-affinity receptors for 1,25-dihydroxyvitamin D3 in human peripheral blood mononuclear cells: Presence in monocytes and induction in T lymphocytes following activation. J. Clin. Endocrinol. Metab..

[69] Maharaj S., Chang S., Kloecker G., Chesney J., Redman R., Rojan A. (2022). Venous and arterial thromboembolism with immunotherapy compared to platinum-based therapy. Thromb. Res..

[70] Dong M., Yu T., Zhang Z., Zhang J., Wang R., Tse G., Liu T., Zhong L. (2022). ICIs-Related Cardiotoxicity in Different Types of Cancer. J. Cardiovasc. Dev. Dis..

[71] Leiter A., Carroll E., De Alwis S., Brooks D., Shimol J.B., Eisenberg E., Wisnivesky J.P., Galsky M.D., Gallagher E.J. (2021). Metabolic disease and adverse events from immune checkpoint inhibitors. Eur. J. Endocrinol..

[72] Michailidou D., Khaki A.R., Morelli M.P., Diamantopoulos L., Singh N., Grivas P. (2021). Association of blood biomarkers and autoimmunity with immune related adverse events in patients with cancer treated with immune checkpoint inhibitors. Sci. Rep..

[73] Akturk H.K., Alkanani A., Zhao Z., Yu L., Michels A.W. (2018). PD-1 Inhibitor Immune-Related Adverse Events in Patients With Preexisting Endocrine Autoimmunity. J. Clin. Endocrinol. Metab..

[74] Menzies A.M., Johnson D.B., Ramanujam S., Atkinson V.G., Wong A.N.M., Park J.J., McQuade J.L., Shoushtari A.N., Tsai K.K., Eroglu Z. (2017). Anti-PD-1 therapy in patients with advanced melanoma and preexisting autoimmune disorders or major toxicity with ipilimumab. Ann. Oncol..

[75] Chennamadhavuni A., Abushahin L., Jin N., Presley C.J., Manne A. (2022). Risk Factors and Biomarkers for Immune-Related Adverse Events: A Practical Guide to Identifying High-Risk Patients and Rechallenging Immune Checkpoint Inhibitors. Front. Immunol..

[76] Jing Y., Zhang Y., Wang J., Li K., Chen X., Heng J., Gao Q., Ye Y., Zhang Z., Liu Y. (2021). Association Between Sex and Immune-Related Adverse Events During Immune Checkpoint Inhibitor Therapy. JNCI J. Natl. Cancer Inst..

[77] Wong S.K., Nebhan C.A., Johnson D.B. (2021). Impact of Patient Age on Clinical Efficacy and Toxicity of Checkpoint Inhibitor Therapy. Front. Immunol..

[78] Asada M., Mikami T., Niimura T., Zamami Y., Uesawa Y., Chuma M., Ishizawa K. (2021). The Risk Factors Associated with Immune Checkpoint Inhibitor-Related Pneumonitis. Oncology.

[79] Sussman T.A., Li H., Hobbs B., Funchain P., McCrae K.R., Khorana A.A. (2021). Incidence of thromboembolism in patients with melanoma on immune checkpoint inhibitor therapy and its adverse association with survival. J. Immunother. Cancer.

[80] Garitaonaindia Y., Blanco M., Franco F., Torrente M., Calvo V., Collazo A., Alba A.G.d., Sanchez J.C., Gutiérrez L., Royuela A. (2022). Risk factors for cardiovascular events in patients treated with immunotherapy. J. Clin. Oncol..

[81] Lutgens E., Seijkens T.T.P. (2020). Cancer patients receiving immune checkpoint inhibitor therapy are at an increased risk for atherosclerotic cardiovascular disease. J. Immunother. Cancer.

[82] Taube J.M., Klein A., Brahmer J.R., Xu H., Pan X., Kim J.H., Chen L., Pardoll D.M., Topalian S.L., Anders R.A. (2014). Association of PD-1, PD-1 ligands, and other features of the tumor immune microenvironment with response to anti-PD-1 therapy. Clin. Cancer Res..

[83] Gautron A.S., Dominguez-Villar M., de Marcken M., Hafler D.A. (2014). Enhanced suppressor function of TIM-3+ FoxP3+ regulatory T cells. Eur. J. Immunol..

[84] Moey M.Y.Y., Tomdio A.N., McCallen J.D., Vaughan L.M., O’Brien K., Naqash A.R., Cherry C., Walker P.R., Carabello B.A. (2020). Characterization of Immune Checkpoint Inhibitor-Related Cardiotoxicity in Lung Cancer Patients from a Rural Setting. JACC CardioOncol.

[85] Quagliariello V., Passariello M., Di Mauro A., Cipullo C., Paccone A., Barbieri A., Palma G., Luciano A., Buccolo S., Bisceglia I. (2022). Immune checkpoint inhibitor therapy increases systemic SDF-1, cardiac DAMPs Fibronectin-EDA, S100/Calgranulin, galectine-3, and NLRP3-MyD88-chemokine pathways. Front. Cardiovasc. Med..

[86] Ruffo E., Wu R.C., Bruno T.C., Workman C.J., Vignali D.A.A. (2019). Lymphocyte-activation gene 3 (LAG3): The next immune checkpoint receptor. Semin. Immunol..

[87] Grabie N., Lichtman A.H., Padera R. (2019). T cell checkpoint regulators in the heart. Cardiovasc. Res..

[88] Lee R.E., Lotze M.T., Skibber J.M., Tucker E., Bonow R.O., Ognibene F.P., Carrasquillo J.A., Shelhamer J.H., Parrillo J.E., Rosenberg S.A. (1989). Cardiorespiratory effects of immunotherapy with interleukin-2. J. Clin. Oncol..

[89] Besler C., Lang D., Urban D., Rommel K.P., von Roeder M., Fengler K., Blazek S., Kandolf R., Klingel K., Thiele H. (2017). Plasma and Cardiac Galectin-3 in Patients with Heart Failure Reflects Both Inflammation and Fibrosis: Implications for Its Use as a Biomarker. Circ. Heart Fail..

[90] Pirozzi F., Poto R., Aran L., Cuomo A., Galdiero M.R., Spadaro G., Abete P., Bonaduce D., Marone G., Tocchetti C.G. (2021). Cardiovascular Toxicity of Immune Checkpoint Inhibitors: Clinical Risk Factors. Curr. Oncol. Rep..

[91] Turk A., Kunej T. (2022). Shared Genetic Risk Factors Between Cancer and Cardiovascular Diseases. Front. Cardiovasc. Med..

[92] Udagawa C., Nakano M.H., Yoshida T., Ohe Y., Kato K., Mushiroda T., Zembutsu H. (2022). Association between genetic variants and the risk of nivolumab-induced immune-related adverse events. Pharmacogenomics.

[93] Subramanian S., Liu C., Aviv A., Ho J.E., Courchesne P., Muntendam P., Larson M.G., Cheng S., Wang T.J., Mehta N.N. (2014). Stromal cell-derived factor 1 as a biomarker of heart failure and mortality risk. Arterioscler. Thromb. Vasc. Biol..

[94] Koene R.J., Prizment A.E., Blaes A., Konety S.H. (2016). Shared Risk Factors in Cardiovascular Disease and Cancer. Circulation.

